# Music and reading activities in early childhood associated with improved language development in preterm infants at 2–3 years of age

**DOI:** 10.3389/fpsyg.2024.1394346

**Published:** 2024-09-11

**Authors:** Kaisamari Kostilainen, Noora Fontell, Kaija Mikkola, Satu Pakarinen, Paula Virtala, Minna Huotilainen, Vineta Fellman, Eino Partanen

**Affiliations:** ^1^Cognitive Brain Research Unit, Faculty of Medicine and Faculty of Educational Sciences, University of Helsinki, Helsinki, Finland; ^2^Centre of Excellence in Music, Mind, Body and Brain, Faculty of Medicine and Faculty of Educational Sciences, University of Helsinki, Helsinki, Finland; ^3^Department of Pediatrics, Pediatric Research Center, Neonatology, New Children’s Hospital, University of Helsinki, Helsinki, Finland; ^4^Helsinki University Hospital, Helsinki, Finland; ^5^Department of Clinical Sciences, Pediatrics, Lund University, Lund, Sweden; ^6^Children’s Hospital, University of Helsinki, Helsinki, Finland

**Keywords:** Bayley scales of infant and toddler development, language development, music playschool, preterm infant, reading, singing

## Abstract

**Introduction:**

Children born preterm are at increased risk for adverse neurodevelopmental outcomes. Music and reading activities in childhood could ameliorate these difficulties, as they have shown benefits on both neural and behavioral levels. However, only a few studies have assessed these potential benefits in preterm-born children. We investigated whether music and language activities in early childhood are associated with improved developmental outcomes of preterm-born children.

**Methods:**

The cognitive, language, and motor skills of 45 children, born between 24 and 34 gestational weeks, were tested at 23–38 months of corrected age with the Bayley Scales of Infant and Toddler Development, Third Edition. Background information, including parental education, and the weekly amount of music and language-related activities was collected using parental questionnaires.

**Results:**

The amount of singing, playing musical instruments and reading aloud was associated with better language skills. Moreover, children who had participated in a music playschool had better language skills when compared to those children who had not participated in a music playschool. Maternal education was associated with music playschool participation and better language and motor skills in children attending music playschool.

**Discussion:**

Interactive music and language activities in early childhood may improve language skills in preterm-born children. Informing and guiding parents at an early stage to integrate these activities into their daily lives could be a one way of supporting the development of preterm-born children.

## 1 Introduction

Preterm birth is a global health problem covering 4–12% of births worldwide (Vogel et al., 2018; Ohuma et al., 2023). Depending on the gestational weeks (GW) at birth, an infant can be born either as moderate to late premature (32–37 GW), very premature (28–32 GW), or extremely premature (<28 GW; World Health Organization, 2020). Preterm birth increases the risk for adverse neurodevelopmental outcome and cognitive deficits in the development of speech and language, including delays in vocabulary and grammatical development, phonological processing, and language comprehension are relatively common in this population (McGowan and Vohr, 2019). While the risk for neurodevelopmental deficits is the highest in very or extremely preterm-born infants (Anderson et al., 2003; Løhaugen et al., 2013; Chung et al., 2020), developmental delays have been increasingly reported also in moderate to late preterm infants, especially in the language domain (Cheong et al., 2017).

Finding means to improve the developmental trajectories of preterm infants is paramount. Formal and informal music activities in childhood may influence brain development in full-term-born children, as there is evidence of the favorable effects of formal musical training (e.g., instrumental lessons) on brain structure and function in children (e.g., Putkinen et al., 2015; Tervaniemi et al., 2018). Informal music activities, i.e., when there is no direct intention to learn specific musical skills, such as music playschool that consists of different musical activities in a small group guided by a trained music educator (e.g., singing, playing, moving with the music, and listening to music), have also been demonstrated to improve auditory processing and linguistic skills in children. For example, Putkinen et al. (2019), demonstrated in their longitudinal study that attendance in a music playschool was connected to improved sound processing in preschool-aged children. Linnavalli et al. (2018), in turn, showed that weekly attendance in a music playschool improved phoneme processing and vocabulary skills in 5–6-year-old children.

Even informal music activities conducted at home, such as singing and playing, have been associated with improved sound processing and attention in 2–3-year-old children (Putkinen et al., 2013). Many researchers now consider that musical interventions or musical activities benefit development, however, not all studies have shown support for the broad benefits of musical activities in child development. The general conclusion in the field is that musical activities provide specific benefits for children’s development although the effects may be narrow (Dumont et al., 2017) and dependent on mediating factors (Schellenberg et al., 2015). However, some recent meta-analyses suggest that the transfer effects of music on cognition are extremely weak (Sala and Gobet, 2020).

Shared reading activities may also influence language development, as it requires both exposure to language and active use or practice of it. Parental interaction may be especially relevant, as the parent focuses the attention of the infant on language while providing support and emotional care (Virtala and Partanen, 2018). Consistent with this, the seminal study of Braid and Bernstein (2015) investigated the impact of book reading on cognitive development in preterm-born children with a large sample size of 1,400 preterm infants born at 22–36 weeks of gestation. Their study demonstrated that reading aloud at least two times per week was associated with improved cognitive skills at 2 years of age. Also, a more recent study by Neri et al. (2021) suggested that parental book-reading implemented already during the neonatal intensive care can support language development of preterm-born infants in the first 2 years of life. Shared reading activities improve language skills also generally in typically developing children as a meta-analysis showed that shared storybook reading offers a rich linguistic experience for the child, as well as promotes the development of vocabulary and supports later reading skills (Flack et al., 2018).

Taken together, research indicates that informal music and reading activities promote cognitive development in typically developing full-term-born children. However, no studies to date have investigated the associations between informal music activities and development of preterm infant, and studies regarding the effects of reading activities on their development is still scarce. In the current study, we examined whether informal music and language-related activities during infancy and early childhood were associated with improved cognitive development in preterm-born children at 2–3 years of age.

## 2 Materials and methods

### 2.1 Participants

Forty-five preterm-born children at 22.9–38.2 months corrected age (mean 28.4 months, SD 4.7) participated in this study. The children were born between 24 and 34 gestational weeks to Finnish-speaking parents and were initially recruited to a longitudinal Singing Kangaroo study while receiving medical care in the neonatal wards of the Helsinki University Central Hospital, Finland (Kostilainen et al., 2021a,b, 2023). As the neonatal parental singing intervention showed no association with Bayley-III scores (Kostilainen et al., 2023), and the music and language-related activities in early childhood between the intervention and control groups did not differ, these groups were combined in this study. During enrollment, the infants were in stable medical condition, with no cerebral hemorrhage stages III-IV, nor congenital central nervous system abnormalities. For the Singing Kangaroo study, only infants born between 24 and 34 gestational weeks were recruited so that the postnatal singing intervention could be conducted for several weeks before term age (Kostilainen et al., 2021a). The participant demographics are presented in Table 1. The study was approved by the Ethics Committee of the Hospital District of Helsinki (Ethics Committee for gynecology and obstetrics, pediatrics, and psychiatry 65/13/03/03/2012). The parents gave their written informed consent to participate after receiving both oral and written information about the study.

**Table 1:** Demographics of participants.

	Mean	SD	Range
*N* = 45 (female 42%)			
Gestational age at birth (weeks), *n* = 44	30.3	2.1	24.7–34.1
Weight at birth (g), *n* = 43	1459.5	398.8	900–2800
Maternal education (years), *n* = 43	17.5	3.1	11–25
Paternal education (years), *n* = 42	15.5	3.4	9–25.3

### 2.2 Data collection

#### 2.2.1 Bayley Scales of Infant and Toddler Development, Third Edition

At 2–3 years of corrected age, the children were assessed with the Bayley Scales of Infant and Toddler Development, Third Edition (Bayley-III; Bayley, 2006). Bayley-III is an extensively used tool for the assessment of early childhood development and early detection of developmental delays. It includes the assessment of five domains: cognition, language, motor, socio-emotional, and adaptive behavior in infants aged from 1 to 42 months. In this study, three major areas of development were evaluated in the following order: cognition, language (including receptive and expressive communication), and motor (including fine and gross motor). The cognitive part included tasks related to sensorimotor development, memory, and concept formation. The language part included word comprehension, the ability to respond to words, preverbal communication, vocabulary, and syntactic development. Motor development was measured with tasks related to functional hand skills, manipulation of objects, static positioning, and movement of limbs and balance.

The Bayley-III assessments were conducted by a research assistant, a student of psychology, under the supervision of a licensed psychologist (EP). The assessments were carried out during one approximately 3-h session (including breaks) in a quiet test room in the Department of Psychology and Logopedics, Faculty of Medicine, University of Helsinki, Finland. In line with the Bayley-III instructions, the parents were permitted to be present, however, they were not allowed to assist the child during the test. In some children, the assessment of all three domains (cognition, language, motor) was not possible, reducing the sample size in those analyses involving all domains (See section Statistical Analyses).

#### 2.2.2 Self-report questionnaire

Background information was collected using self-report questionnaires, filled in by the parents (*n* = 44) during the child’s Bayley-III test. In addition to questions concerning basic information (e.g., parental education), parents were asked to evaluate how much children were currently exposed to music and language activities weekly, and whether children had participated in a music playschool. According to our hypotheses, six main factors from the questionnaire were chosen for the analyses (Table 2).

**Table 2:** The six main factors from the self-report questionnaires used in the analyses.

Factors
Mother’s education (years in total)
Father’s education (years in total)
Attendance in a music playschool (yes/no) If yes:
at what age (e.g., 6–12 months)
for how long (e.g., 6 months)
how often (e.g., 1 h/week)
Music and language-related activities in the same space with the child currently (hours per week, music playschool activities excluded)
playing live music
singing
listening to music
reading aloud
Language-related activities together with the child currently (rated 1–4*, music playschool activities excluded)
singing
reading nursery rhymes or books
Music activities currently (1–4^*^, music playschool activities excluded)
playing instruments together with the child
the child playing instruments alone

* 1 = almost never, 2 = once a month at most, 3 = several times per month, 4 = several times per week.

### 2.3 Statistical analyses

The statistical analyses were conducted using SPSS 28 (IBM Corporation, NY, United States). Bayley-III age-standardized index scores were used in the analyses. Missing values were not replaced. To reduce the number of multiple comparisons, sum and mean scores of theoretically related background variables; music and language-related activities in the same space with the child, language-related activities together with the child, and music activities were used instead of scores from several individual items (Table 3). Multiple linear regression was used to examine the associations between the Bayley-III scores, music/language activities, and mother’s education years. In the models, the dependent variable (cognitive, language and motor scores) was regressed on predicting variables of music and language activities and mothers’ education years. Furthermore, the impact of music playschool on the Bayley performance was tested using the repeated-measures Analysis of variance (rmANOVA) with music playschool participation as a between-group factor and Bayley scores (cognitive, language, and motor) as within-group factors. Only those children who had all Bayley scores measured (cognitive, language, and motor) were included in the analysis (n = 37). The data were further analyzed with repeated-measures Analysis of covariance (rmANCOVA) to assess the possible effect of maternal education on Bayley scores in children that had attended music playschool.

**Table 3:** Variables generated from the background questionnaire and used in the analyses.

Variable changes
Music and language-related activities in the same space with the child currently (hours per week) Sum variable: playing live music + listening to music singing + reading aloud Language-related activities together with the child currently (1–4^*^) Mean variable: singing + reading nursery rhymes or books Music activities currently (mean from 1 to 4^*^) Mean variable: playing instruments together with the child + the child playing instruments alone

* 1 = almost never, 2 = once a month at most, 3 = several times per month, 4 = several times per week.

The possible effect of sex on the Bayley scores was tested using sex (male, female) as a between-group factor and Bayley scores (cognitive, language, and motor) as within-group factors. A Greenhouse–Geisser correction was used if the assumption of sphericity was not met, and Bonferroni correction was used in the *post hoc* tests. Partial eta squared (η^2^) is used when reporting effect sizes for rmANOVA analyses. Non-parametric Mann–Whitney U-tests were used when comparing the background information of the divided groups (children in music playschool vs. children not in music playschool).

## 3 Results

The mean index and subtest scores of all participants on Bayley-III scales are presented in Table 4 and the amount of weekly music and language-related activities is reported in Table 5. No associations between the mothers’ and fathers’ education years in total and the children’s Bayley scores were found (*p* > 0.08). The gestational age at birth was not associated with the Bayley scores, respectively. Also, no sex differences were found in the Bayley scores.

**Table 4:** The mean Bayley-III index and subtest scores for all participants (not all participants completed all the subtests).

	Mean	SD	Range
Bayley-III index score			
Cognitive, *n* = 45	101.9	10	85–125
Language, *n* = 43	107.6	11.3	83–132
Motor, *n* = 40	104	11.8	79–136
Bayley-III subtest score			
Cognitive	10.4	2.0	7–15
Receptive language	12.1	2.1	7–16
Expressive language	10.3	2.9	4–15
Fine motor	10.7	2.0	7–16
Gross motor	10.5	2.6	5–18

**Table 5:** The questionnaire results regarding the amount of weekly music and language-related exposure in the families.

	Mean	SD	Range
Music and language-related activities in the same space with the child (hours per week)			
playing live music	0.7	1.3	0–7
singing	3.3	3.1	0–10
listening to music	5.5	3.8	0–15
reading aloud	4.3	3.2	1–15

Twenty-six children (58%, one child’s data missing) had participated or were currently still attending a music playschool. The families had started the activity when their infants were on average 8.5 months of age (range 1–24 months), and music playschool was attended on average 52 min per week (range 30–90 min) for an overall period of 12.3 months (range 2–30 months). When comparing the background information of the divided groups (children in music playschool vs. children not in music playschool), no differences were found (*p* > 0.319 for all comparisons) except for maternal education, U = 94.50, z = −3.225, *p* = 0.001, as the mothers of the children in the music playschool had more education years (Md = 18.00, *n* = 25) than those mothers of the children that had not participated in a music playschool (Md = 15.66, *n* = 18). There were no statistically significant differences in the amount of weekly music and language-related activities between the children who participated in music playschool and who did not participate in music playschool (Supplementary Table 1).

### 3.1 The associations between the Bayley-III scores and the music and reading activities

**Figure 1:**
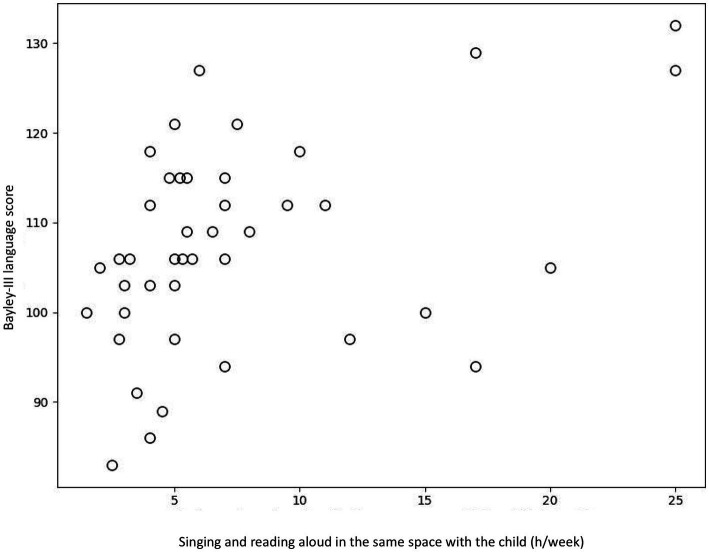
Scatterplot illustrating the association between Bayley-III language score and the amount of singing and reading aloud in the same space with the child (*p* = 0.012).

**Figure 2:**
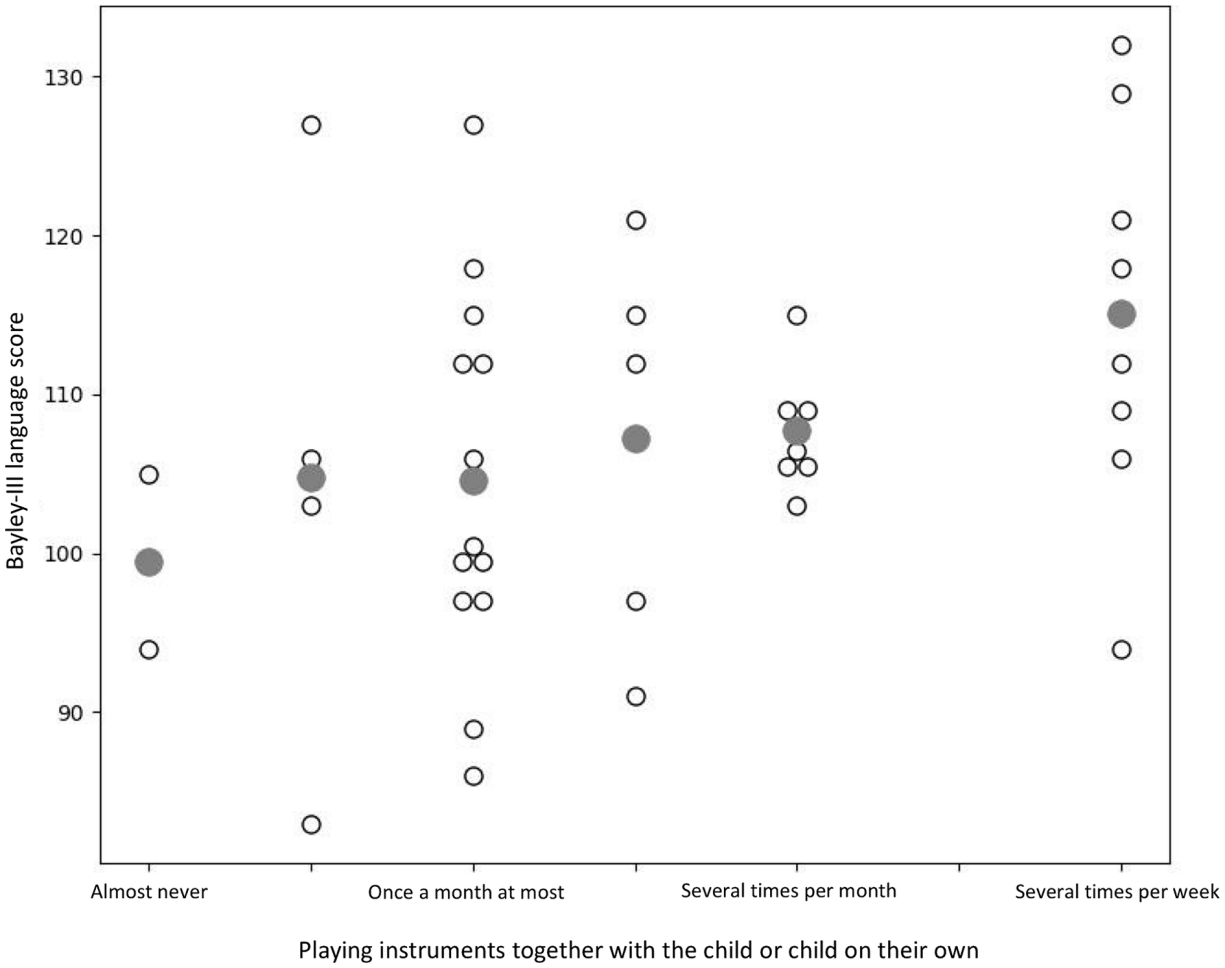
Scatterplot illustrating the association between Bayley-III language score and the amount of playing instruments together with the child or child playing on their own (*p* = 0.013). Grey colored symbol indicates mean value.

The multiple regression analysis showed that the music and language-related activities and mother’s education years did not predict Bayley-III cognitive (*p* = 0.356) nor motor scores (*p* = 0.318). However, the multiple regression model with music and language-related activities and mother’s education years predicting Bayley-III language scores resulted in a significant model *F*(5, 34) = 3.724, *p* = 0.009, *R^2^* = 0.354. When examining the individual predictors, we found that singing and reading aloud in the same space with the child (t = 2.647, *p* = 0.012; Figure 1) as well as playing instruments together with the child or child on their own (t = 2.611, *p* = 0.013; Figure 2) were significant predictors for Bayley-III language scores. On the contrary, mother’s education years did not predict children’s language scores (t = 1.346, *p* = 0.187). The regression table of the significant model is presented in Table 6.

**Table 6:** The regression coefficients, statistical significances, and R-squared values of mother’s education years and music and reading activities on Bayley-III language scores.

Variable	*B*	*SE B*	β	*t*	*p*
Mother’s education years	0.811	0.603	0.217	1.346	0.187
Playing live music and listening to music in the same space with the child	–0.355	0.445	–0.122	–0.797	0.431
Singing and reading aloud in the same space with the child	0.879	0.332	0.418	2.647	0.012*
Singing and reading nursery rhymes or books together with the child	–5.518	6.008	–0.165	–0.919	0.365
Playing instruments together with the child or the child playing instruments alone	5.772	2.211	0.458	2.611	0.013*
R^2^	0.354				

* *p* < 0.05.

**Figure 3:**
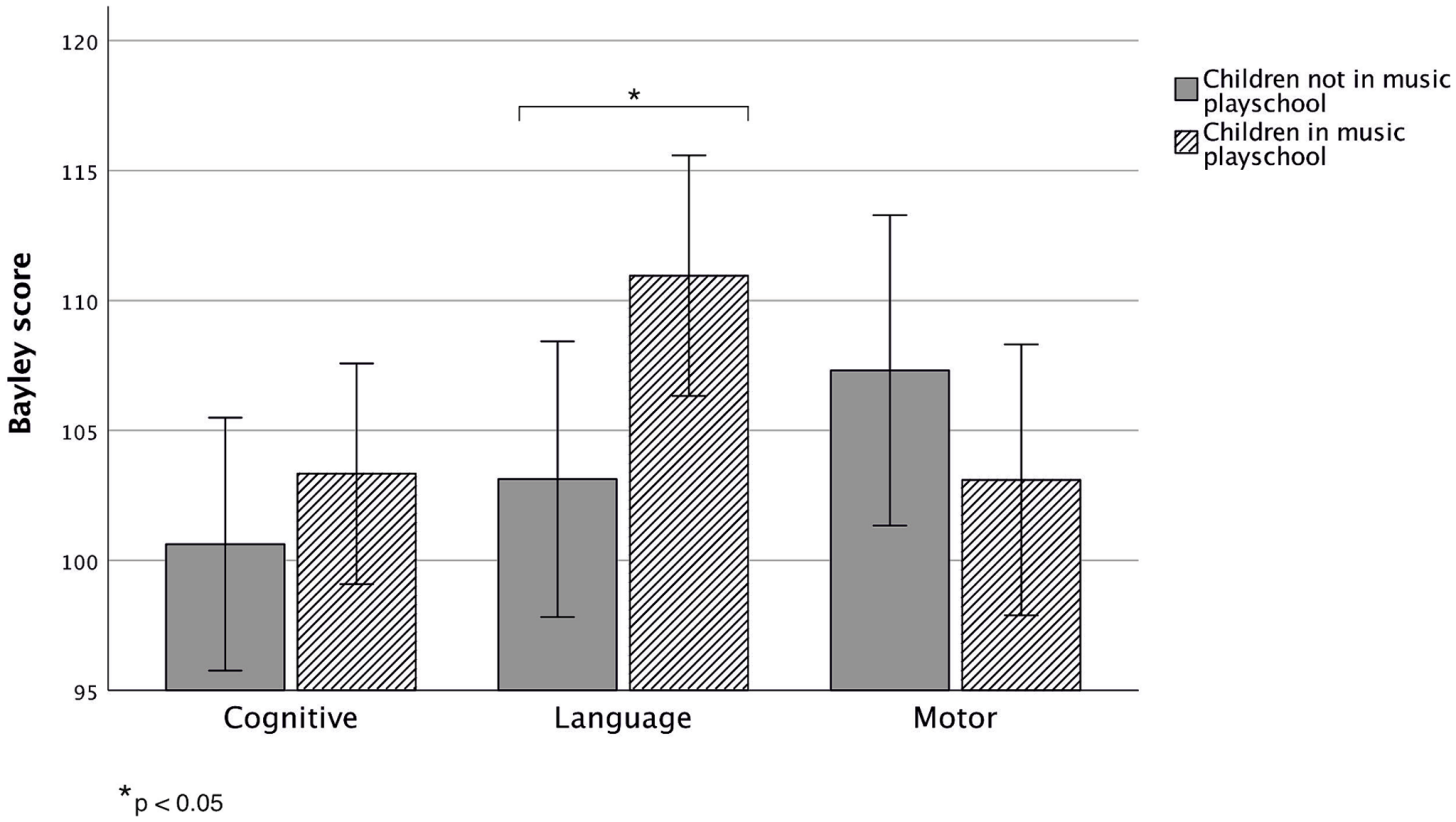
Bayley cognitive, language, and motor mean scores with 95% confidence intervals between children who had not participated in a music playschool (grey) and children who had participated in a music playschool (grey/white). A statistically significant group difference (*p* = 0.03) was found in the Bayley language performance between the two groups when maternal education was not controlled for (cognitive: *p* = 0.40; motor: *p* = 0.29).

When examining the effect of music playschool participation on Bayley cognitive, language, and motor scores, rmANOVA showed an interaction effect of music playschool participation and Bayley scores, *F*(2, 70) = 3.433, *p* = 0.038, *η*^2^ = 0.089. This result was due to children who had participated in a music playschool having statistically higher Bayley language scores than those children who did not participate in a music playschool, *p* = 0.03 (Figure 3). However, as the results showed a group difference in maternal education, an additional analysis was conducted while adjusting for maternal education. The analysis revealed a main effect of the covariate ‘Maternal education’ *F*(1, 33) = 5.138, *p* = 0.03, *η*^2^ = 0.135, meaning that maternal education was positively associated with the Bayley scores. Also, when controlling for the confounding factor ‘Maternal education’, the group difference did not remain statistically significant, *F*(2, 66) = 2.769, *p* = 0.07, *η*^2^ = 0.077. After a more detailed correlation analysis, the results revealed that higher maternal education was positively associated with higher Bayley language scores, r_s_ = 0.436, *n* = 23, *p* = 0.037, and motor scores, r_s_ = 0.472, *n* = 22, *p* = 0.026, in the music playschool group children. Also, after evaluating the correlation between maternal education and the amount of the music and language-related activities in general, higher maternal education was found to be positively associated with how much there was reading aloud in the same space with the child, r_s_ = 0.305, *n* = 43, *p* = 0.47.

## 4 Discussion

In this study, we examined the association of music and language-related activities during early childhood on cognitive development in preterm-born children at 2–3 years of age. The results showed that regular interactive music and language-related activities, such as singing and reading aloud as well as playing musical instruments, were associated with improved Bayley language skills. Hence, interactive music and language-activities may support language development in preterm-born children.

On average, the preterm children in this study performed similarly as their age equivalent full-term peers (Serenius et al., 2013). In our data, interactive music, and language-related activities, such as singing and reading in the same space and together with the child were associated with better Bayley-III language scores unlike passive music activities, such as music listening. Social interaction is known to be an important factor in speech learning (Kuhl et al., 2003; Kuhl, 2004; Lytle et al., 2018) and interactive music-making in infancy and early childhood has been shown to improve language development more efficiently than passive music listening. For example, in a study by Gerry et al. (2012), 6-months-of-age full-term born infants and their parents were randomly assigned either to active music classes consisting of movement, singing, and instrument playing or passive music classes consisting of music listening once a week for 6 months. After the intervention period, infants in the active music classes showed better prelinguistic communicative gestures and social behavior when compared to infants in the passive music group (Gerry et al., 2012). Thus, social interaction is an important factor in music activities enhancing both language and social development.

Musical interaction between the caretaker and the child has been proposed to have benefits for child development (Virtala and Partanen, 2018). Consistent with the idea that benefits of musical activities arise from reciprocal activity incorporating music, music playschool participation together with the caregiver in our study was associated with improved language skills. Music playschool offers moments of musical interaction in a positive environment. Hence, it may be that both the reciprocal nature of music playschool and the positive environment together improved language skills in our study. However, this result should be handled with extra caution as maternal education affected the results and the impact of music playschool on preterm infant development should be assessed more closely in future studies.

As our results indicated, participation in a music playschool with their preterm-born child became more likely with increasing maternal education. It may be that higher-educated parents have more resources, such as money and knowledge, to offer their children developmentally supportive activities. For example, parents can afford paid activities such as music playschool, or parents read more to their children as in this study and the study reviewed earlier by Braid and Bernstein (2015), where the amount of shared reading was associated with enhanced cognitive development in preterm infants, maternal education was associated with how much parents read to their children. In our study, higher maternal education level was also associated with higher Bayley language and motor scores in those children who had participated or were still participating in a music playschool.

Maternal education is known to be closely related to child development, and overall, the socioeconomic status (SES) of parents has been shown to impact language development. For example, Rowe (2008) investigated the connections between the speaking styles of parents from different socioeconomic backgrounds. They found that parents from low and high SES backgrounds did not differ in their verbal ability, but they differed in general knowledge of child development and the importance of child-directed speech. Hence, the parents from high SES backgrounds had more word diversity, they used “parentese” (speaking style with exaggerated phonetic patterns) when talking to their child and talked more in general (Rowe, 2008). Thus, parents with higher education levels have more knowledge of child development, which affects how they interact with their children. This likely explains why mothers with more education years placed their children in music playschools in our data. In future studies, it would be important to examine more thoroughly what different factors, such as music, mother’s education, and interactive behavior contribute to the benefits of music activities.

As parents are usually the primary source of language input for their children, educating parents of preterm infants from different education and socioeconomic backgrounds about the benefits of developmentally supportive activities, such as reading aloud and using music activities in early childhood, is recommended. Offering parental psychoeducation is of value, however, parents from lower education level should also be offered resources to support child development, for example, by increasing their possibilities to attend music playschool. Even if increasing resources would not be possible, distributing knowledge can be beneficial as the study by Suskind et al. (2015) demonstrated that educating mothers from low SES backgrounds about the importance of their language input can increase knowledge, have an impact on mothers’ behavior, and change the home language environment. Parental education, as in educating parents to behave and interact in a manner that supports child development (McMahon et al., 2012) can also influence parental sensitivity, which has been shown to enhance language learning (Madigan et al., 2019). Based on these results, future studies examining the effects of both parent education and music playschool participation on cognitive development in preterm infants, especially from low-SES families would be needed.

### 4.1 Limitations

The children were originally planned to be assessed with Bayley-III at 24 months of corrected age (± 3 months). Due to restrictions in assessment schedules due to the COVID-19 pandemic, some of the children were assessed later than initially planned. Bayley-III being an age-standardized test, it was possible to conclude the assessments despite part of the children exceeding the age limit set for the measurements. In this study, the amount of music and language-related activities was examined using self-report questionnaires answered by the parents. Hence, information about the specific contents and durations of the reading and music playschool activities during the first 2 years of age could not be assessed. Also, as no information on the amount of parent–child interactions other than music or reading was collected, we cannot exclude the possibility that other interactive activities may have affected the results. Moreover, as we studied infants born extremely and very preterm, we cannot draw further conclusions from these results regarding infants born moderate to late preterm.

It should be considered that the number of participants in this study was limited and did not include a control group of full-term children. In addition, parents participating in studies are often highly educated, as in this study. Therefore, it would be important to find ways to involve families from different socioeconomic backgrounds. Future studies should consider the use of more systematic and controlled early childhood interventions and outcome measures with higher number of participants and RCT designs, including the SES of the family at randomization.

## 5 Conclusion

Regular interactive music and language-related activities in early childhood, such as singing, reading aloud, and playing musical instruments may improve language skills in preterm-born children. Informing and guiding parents from early on how to utilize these developmentally supportive interactive activities in their daily lives might be one potential means to support preterm infant’s development. Such instructions could form a basis for early music and language-related intervention that would be feasible and cost-effective for parents from different educational and SES backgrounds to conduct.

## Data availability statement

The raw data supporting the conclusions of this article will be made available by the authors, without undue reservation.

## Ethics statement

The studies involving humans were approved by Ethics Committee of the Hospital District of Helsinki. The studies were conducted in accordance with the local legislation and institutional requirements. Written informed consent for participation in this study was provided by the participants’ legal guardians/next of kin.

## Author contributions

KK: Formal analysis, Visualization, Writing – original draft, Writing – review & editing, Funding acquisition, Methodology, Validation. NF: Methodology, Writing – review & editing. KM: Conceptualization, Writing – review & editing, Data curation, Project administration, Supervision. SP: Conceptualization, Writing – review & editing, Methodology, Supervision. PV: Supervision, Writing – review & editing. MH: Conceptualization, Supervision, Writing – review & editing, Funding acquisition, Methodology, Project administration. VF: Conceptualization, Funding acquisition, Supervision, Writing – review & editing, Project administration. EP: Conceptualization, Supervision, Writing – review & editing, Methodology, Project administration.
