# Supplementary material for: Music and reading activities in early childhood associated with improved language development in preterm infants at 2–3 years of age
Source: Front Psychol. 2024 Sep 11;15:1394346. doi: 10.3389/fpsyg.2024.1394346 (PMC11423423; doi:10.3389/fpsyg.2024.1394346)
Supplement: Supplementary file 1 [file Table_1.DOCX]

**Supplementary Table 1**. The amount of weekly music and language-related exposure (mean, standard deviation, range) in the families whose children were in music playschool and who were not in music playschool.

|  | Children in music playschool (n = 26) | Children not in  music playschool (n = 18) | P value |
| --- | --- | --- | --- |
| Music and language-related activities in the same space with the child (hours per week) |  |  |  |
| playing live music | 0.5 (0.8)  0–3 | 1.0 (1.8)  0–7 | 0.427 |
| singing | 3.7 (3.4)  1–10 | 2.5 (2.5)  0–10 | 0.318 |
| listening to music | 6.0 (4.1)  0–15 | 4.8 (3.5)  0–12 | 0.387 |
| reading aloud | 4.6 (3.1)  1–15 | 3.9 (3.3)  1–15 | 0.187 |
